# Genes Critical for Developing Periodontitis: Lessons from Mouse Models

**DOI:** 10.3389/fimmu.2017.01395

**Published:** 2017-10-27

**Authors:** Teun J. de Vries, Stefano Andreotta, Bruno G. Loos, Elena A. Nicu

**Affiliations:** ^1^Department of Periodontology, Academic Centre for Dentistry Amsterdam (ACTA), University of Amsterdam, VU University Amsterdam, Amsterdam, Netherlands; ^2^Opris Dent SRL, Sibiu, Sibiu, Romania

**Keywords:** periodontitis, mouse models, immune modulation, osteoclast, bone resorption, chronic periodontitis, knockout mouse, transgenic mice

## Abstract

Since the etiology of periodontitis in humans is not fully understood, genetic mouse models may pinpoint indispensable genes for optimal immunological protection of the periodontium against tissue destruction. This review describes the current knowledge of genes that are involved for a proper maintenance of a healthy periodontium in mice. Null mutations of genes required for leukocyte cell–cell recognition and extravasation (e.g., *Icam-1, P-selectin, Beta2-integrin/Cd18*), for pathogen recognition and killing (e.g., *Tlr2, Tlr4, Lamp-2*), immune modulatory molecules (e.g., *Cxcr2, Ccr4, IL-10, Opg, IL1RA, Tnf-*α *receptor, IL-17 receptor, Socs3, Foxo1*), and proteolytic enzymes (e.g., *Mmp8, Plasmin*) cause periodontitis, most likely due to an inefficient clearance of bacteria and bacterial products. Several mechanisms resulting in periodontitis can be recognized: (1) inefficient bacterial control by the polymorphonuclear neutrophils (defective migration, killing), (2) inadequate antigen presentation by dendritic cells, or (3) exaggerated production of pro-inflammatory cytokines. In all these cases, the local immune reaction is skewed toward a Th1/Th17 (and insufficient activation of the Th2/Treg) with subsequent osteoclast activation. Finally, genotypes are described that protect the mice from periodontitis: the SCID mouse, and mice lacking *Tlr2/Tlr4*, the *Ccr1/Ccr5*, the *Tnf-*α *receptor p55*, and *Cathepsin K* by attenuating the inflammatory reaction and the osteoclastogenic response.

## Introduction

Periodontitis is a destructive bacterial-induced chronic inflammatory disease of the tooth-supporting tissues that leads to tooth loss due to resorption of the tooth surrounding connective tissues and alveolar bone if not properly and timely treated. The biological complexity of human periodontitis is highly comparable to other chronic immune disorders (CIDs) where multiple factors determine the resultant immune fitness (1). In this way, periodontitis is related to an aberrant immune response to the bacterial biofilm on the teeth and tooth roots that border the periodontal tissues (2, 3). Most people live in symbiosis with their oral microbiome and specifically with a thin layer of dental plaque on the teeth. These individuals present some sort of immunological tolerance. The most prominent immune cell that is constantly present in the gingival dental plaque interface is the PMN, with about 30,000 of them per minute extravasating into the gingival crevices around the teeth. They have a tolerant and non-hyperreactive phenotype, not producing pro-inflammatory signals, resulting in maintenance of periodontal health in the potentially “dangerous” oral environment harboring billions of bacteria, including low level (dormant) potential bacterial pathogens. Moreover, dendritic cells home to lymphoid organs and nodes for “training” the host in tolerance, but also preparing for adaptive immunity when needed. In fact, the dendritic cells steer the development of T regulatory cell (Treg), to be found in the gingiva. Inside the gingival tissues one can appreciate also a tolerance of B cells (again modulated by Treg), macrophages, as well as fibroblasts, having all the non-reactive host defense imprint. We could compare this state of health or “normality” of the mucosal immunity in the gingiva with that of the other mucosal surfaces, like intestinal mucosal linings, normally not reacting with overt inflammation to bacterial products and food-related antigens (1, 4).

A clear limitation of studying periodontitis in humans is the complexity of the disease, involving interactions between genes, life styles, and the tooth-related microbiome composition. In order to exclude this “noise” between individuals, one can make advantage of mouse models, where inbred strains overcome genetic variations.

Wild-type mice are relatively well protected against periodontitis. Apart from a few reports (5), spontaneous or bacteria-induced periodontitis is scarcely reported. This review describes the emerging field of genetically modified mouse models that develop periodontitis. Since single gene null mutations may already cause periodontitis, mouse knockout models are advantageous in identifying genes that are essential in the protection against periodontitis. Although a challenge with periodontitis-associated bacteria such as *Aggregatibacter actinomycetemcomitans* or *Porphyromonas gingivalis* is often required for periodontitis induction in mice, periodontitis can also occur without exogenous inoculation but in the presence of naturally present bacteria, such as described by Beertsen et al. (6) or even under sterile conditions, such as described by Sheng et al. (7). All ligature-induced periodontitis mouse models are excluded from this review, since we regard this *a priori* wounding of the periodontium as an artificial model that does not reflect natural periodontitis initiation and progression. Where possible, the mouse model findings will be related to human “naturally occurring” genetic mutations.

## Mouse Periodontium, Mouse Periodontitis

The two soft tissue components of the periodontium (From Greek, peri = around, odontos = tooth) are the gingiva and the periodontal ligament. A major difference between the human and mouse dentition is the orientation of the four continuously erupting incisors that are located underneath the mandibular or superior to the maxillary molar arch. Apart from the four incisors, mice have three molars (M1–M3) per quadrant, decreasing in size from front to back. Under normal conditions, the attachment point of the junctional epithelium (i.e., the deeper part of the epithelium that connects gingiva to teeth) is terminating at the cementum-enamel-junction (CEJ) of the teeth.

**Figure 1:**
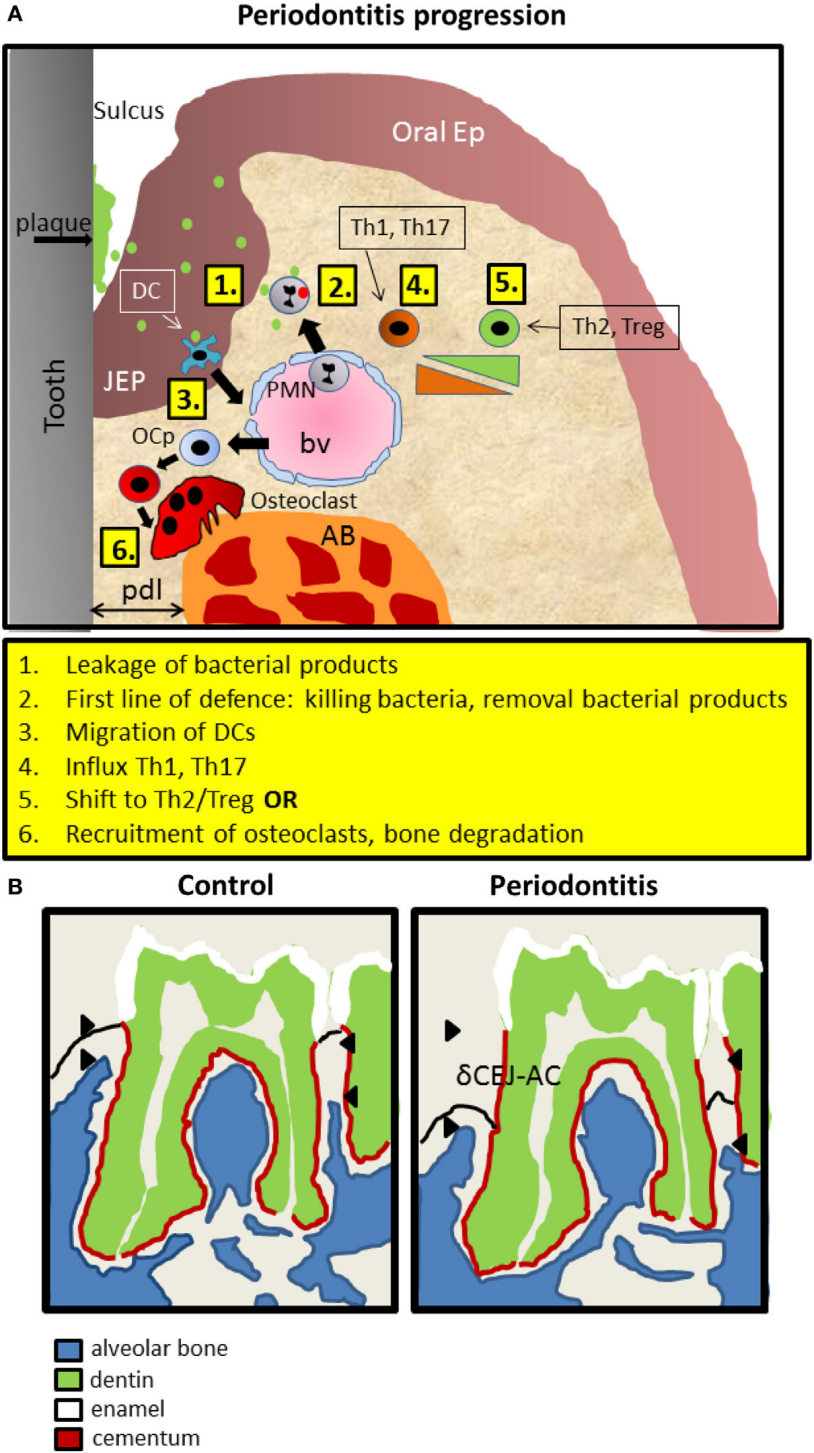
Introduction into mouse periodontitis. **(A)** The sequence of events leading to periodontitis. **1**. Bacterial pressure (green; red inactivated) colonizing the tooth area (plaque) and present in the space between the tooth surface and the epithelium (sulcus), causes the JEP to thicken and retract. 2. First line of defense such as polymorphonuclear neutrophils (PMNs) are attracted to the infection, extravasate out of blood vessels (bv) into the tissue, and kill bacteria and remove bacterial products. 3. Langerhans cells, special dendritic cells within epithelium (ep), recognize bacterial products, migrate away and elicit a Th1 response that is present during early inflammation, coincidental with inflammatory mediators such as IL1, TNF-α, and IL-6. 4. Gradually, in a sustained inflammation, this shifts toward a Th2 phenotype with more anti-inflammatory mediators. Experimental evidence for this sequence was demonstrated by Araujo-Pires et al. (8), Arizon et al. (17), and Bittner-Eddy et al. (18). 5. Finally, and archetypal for periodontitis, precursor cells of osteoclasts (OCp) migrate to the alveolar bone (AB) and differentiate into bone degrading multinucleated osteoclasts (OC). pdl, periodontal ligament; JEP, junctional epithelium; Ep, epithelium; bv, blood vessel. **(B)** Schematic drawing of mouse periodontitis with emphasis on the hard tissues. Redrawn from a microCT image taken from the study by Koide et al. (60). Shown are the full first and part of the second of the three mouse molars of a mandible. The distance between the cementum-enamel-junction and the AB crest (δCEJ-AC) is an objective criterion for establishing periodontitis. This distance increases in periodontitis due to the degradation of AB by OC. The imaginary epithelial border—not visible with microCT is indicated with a black line.

In periodontitis, typically, apical migration of junctional epithelium occurs, concomitant with invasion of inflammatory cells in the gingiva and the epithelial layer and finally recruitment of osteoclasts that degrade underlying alveolar bone. A recent study (8) has refined the sequential influxes of immune cells during periodontitis progression in mice (Figure 1A). Upon an exogenous challenge with periodontitis-associated bacteria, a first line of defense invasion of polymorphonuclear neutrophils (PMNs) will inactivate most bacteria. This is followed by primarily T helper (Th) 1 and Th17 cells, which are replaced gradually by Th2 and Tregs. Ultimately, osteoclasts are expanding at the alveolar bone crest where alveolar bone is been degraded in the end stage of periodontitis (Figure 1A). The widely used objective criterion for the diagnosis of periodontitis, both for men and mice, is the pathologically increased distance between the cementum-enamel-junction (CEJ) and the tip of the alveolar bone. In mice, this distance is measured only at the molar block and increases during periodontitis progression, eventually leading to tooth loss (Figure 1B). It should be emphasized at this stage, that many genes and cell types discussed here, may play a dual role. For instance, PMNs can both prevent periodontitis initiation and progression by timely eliminating bacteria. Alternatively, when not effective, the presence of PMNs can be detrimental for the periodontal tissues, since endured cytokine expression may evoke soft and hard tissue degradation by fibroblasts or inflammatory cells and osteoclasts, respectively.

## Mouse Strains and Periodontitis Susceptibility—Early Observations

Periodontitis and subsequent tooth loss, can occur spontaneously in mice; however, this is a rare phenomenon. Nevertheless, periodontitis can be induced experimentally *via* oral lavage with microbial species that are strongly associated with the development of periodontitis in human [e.g., Ref. (9)] or can be a result of genetic mutations, either or not using bacterial pressure to induce it [e.g., Ref. (10)]. Baker et al. (11) showed that the various mouse strains differ in their susceptibility to develop experimentally induced periodontitis. In these experiments, mice were pre-treated with antibiotics before being infected with viable *P. gingivalis*. Even under a high exposure to *P. gingivalis*, five out of nine inbred strains that were analyzed (A/J, A/HEJ, 129/J, SJL/J, C57BL/6J) were resistant to alveolar bone loss (measured as the distance AC-CEJ, see Figure 1B for explanation) and four were susceptible (AKR/J, DBA/2J, BALB/cByJ, BALB/cJ). In a recent study, this was confirmed by Shusterman et al. (12), who found BALB/c mice to be susceptible for periodontitis and not DBA/2J, C57BL/6J, and A/J mice. This could well explain why until recently periodontitis in mice was hardly encountered, since most genetically modified mice described in this paper originated from the relatively resistant C57BL/6J mice.

## Periodontitis in Mice That Lack Defined Subsets of Immune Cells

### B and T Cells

In periodontal research, the first immune-deficient murine model was introduced by Baker and coworkers in 1994 (13) with the goal to evaluate the effect of *P. gingivalis* infection in “severe combined immune deficiency mice,” the SCID mice. The SCID mouse lacks both T and B lymphocytes. Baker et al. compared two genetically disparate strains of immunocompetent mice, C57BL/6J and BALB/cByJ with an immunodeficient strain: C.B17-*scid*/SzDcr. A pretreatment phase with antibiotics with the attempt to suppress the commensal microflora was followed by an oral infection with *P. gingivalis via* oral lavage for 42 days. Infection with *P. gingivalis* induced alveolar bone loss in immunocompetent and immunodeficient mice, but the degree of alveolar bone loss in immunocompetent strains, BALB/cByJ, was higher than that in the genetically closely related SCID strain. This study indirectly showed that mice with intact T and B cell repertoire display more bone destruction, signifying that immune cells contribute and are necessary for the onset of periodontitis-like bone resorption. In a later report, the same group (14) investigated under the same experimental conditions the role of the T cells using β_2_ m-knockout mice (deficient in CD8^+^ and NK1^+^ T cells), A_β_-knockout mice (fail to generate CD4^+^ T cells), interferon-γ deficient mice and interleukin-6 (IL-6) deficient mice. This study showed that CD4^+^ T cells promote alveolar bone loss, whereas CD8^+^ and NK1^+^ T cells did not play a direct or indirect role in the bone resorption process. A_β_-knockout mice did not demonstrate significant alveolar bone loss when infected with *P. gingivalis*. These studies suggest the possible involvement of certain immune cells, with the obvious caveat that it may not reflect the progressive stages of periodontitis, since this disease is characterized with a sequential influx of defined immune cells over time. Upon infection, and as periodontitis progresses, mouse periodontium is first invaded by Th1 T-cells, followed by Th17 and at the end stage, probably reflecting a more chronic diseased state, Th2 and Tregs invade the soft periodontium (8).

### Dendritic Cells

Dendritic cells are highly specialized innate immune cells that orchestrate the adaptive immune responses. In their immature state, dendritic cells can efficiently capture and process microbial antigens, but as they mature, their phenotype changes, and mature DCs can migrate toward lymphoid organs and prime naïve T cells (15). Human and murine gingiva contains several subsets of DCs, of which the Langerhans cells present in the epithelial compartment are the most studied (16).

Arizon and coworkers employed a mouse model of Langerhans cell-ablation followed by oral inoculation of *P. gingivalis* (17). In this inducible murine *Langerin* knockout model, dendritic cell ablation led to an aggravated local inflammation in the periodontium and more alveolar bone loss. Specifically, in the absence of dendritic cells, a marked increase in the number of B cells and CD4 T cells, together with a lower number of Treg cells, was observed in the inflamed periodontium. Many of the infiltrating T cells, NK cells, or γδ cells expressed also the osteoclast-activating cytokine receptor activator of nuclear factor-κB ligand (RANKL), linking the intense inflammation with the alveolar bone loss. The interpretation of these results was recently questioned by Bittner-Eddy and coworkers, in a different model of Langerhans cell ablation (18). Their model resulted in the targeted ablation of exclusively the Langerhans cells, but left other DC types unaffected such as the Langerin^+^ DCs and the CD8^+^ lymphoid-resident DCs; this is unlike the murine *Langerin*-DTR model employed by Arizon et al. (17). It is this combined deficiency in Langerhans cells and Langerin^+^ DCs that explains the more severe periodontitis in the murine Langerin-model. These mice fail to induce both Th17 and Treg cells, which pushes the phenotype toward a skewed Th1 response and IFN-γ-induced osteoclastogenesis with alveolar bone loss as consequence (18).

Dendritic cell functions are regulated by transcription factors, including forkhead box-O1, *Foxo1*. FOXO1 regulates dendritic cell migration to lymph nodes and lipopolysaccharide (LPS)-induced cytokine expression by dendritic cells. Targeted deletion of *Foxo1* in the dendritic cells has been studied in a mouse model, in which periodontitis was induced by oral inoculation with *P. gingivalis* and *Fusobacterium nucleatum* (19). *Foxo1* deletion resulted in reduced migration of dendritic cells in the epithelium and conjunctive tissue around teeth. These dendritic cells expressed less IL-12 in response to *P. gingivalis* than control mice. The alveolar bone loss was more severe in the mice with a *Foxo1* deletion, probably *via* an increased production of the pro-osteoclastogenic IL-1β, IL-17, and RANKL and insufficient stimulation of B cells by the DCs in the lymph nodes. This latter suggestion is consistent with the findings of Mkonyi et al. in their blocked lymphangiogenesis model; they report that a reduced B-cell activation leads to a compensatory increase in IL-1β and IL-17, and results in enhanced bacteria-induced bone loss (20).

### Macrophages

Analysis of the role of macrophages in periodontitis revealed that the M1 macrophage accumulates and is the predominant macrophage in the periodontium of mice infected with *P. gingivalis*. This coincided with increased levels of pro-osteoclastogenic and inflammatory cytokines IL-1 and IL-6. Mice in which macrophages were depleted with clodronate containing liposomes, were protected from developing periodontitis (21). Since cells from the monocyte/macrophage lineage are also precursors for bone degrading osteoclasts, this finding could suggest that macrophage depletion in turn diminishes osteoclast precursor cells.

## Mouse Models of Genes Associated with the Inflammatory Response to Microbial Pressure

The availability of knockout mice has accelerated mouse periodontitis research. In general, oral gavage models using periodontitis-associated pathogens were needed to induce periodontitis. Apparently, in most cases, exogenous bacterial pressure in conjunction with a loss of function of a certain gene is needed to evoke periodontitis. This way, essential genes necessary for combating the bacterial pressure could be identified. These include those (i) engaged in the adhesion and subsequent extravasation of leukocytes toward the infection area such as selectins and integrins, (ii) genes that are involved in recognition and clearance of bacteria such as Toll-like receptors (*Tlr2, Tlr4*) and the lysosome-associated membrane proteins (*Lamps*), and *Lactoferrin* (iii) modulatory cytokines such as *IL-17* or inflammation inhibitory cytokines. As result of an enduring inflammatory response, proteases are predominantly present in the periodontium, leading to softening of the tissue and to bone degradation. In (iv), the protease models of *Mmp-8, Plasminogen*, and *Cathepsin K* are discussed. In (v), periodontitis models of the structural mutations of the periodontium involving bone, cementum and dentin matrix proteins and mice lacking lymphatics are briefly discussed. Finally (vi), mouse models for periodontitis in conjunction with other inflammatory diseases such as atherosclerosis and rheumatoid arthritis are discussed.

### Adhesion Molecules: Selectins and Integrins

Endothelial and leukocyte adhesion molecules are responsible for the extravasation process that occurs when leukocytes are recruited to the inflammatory site. Upon extravasation, cell–cell and cell–matrix adhesion molecules are required for the homing process of the leukocytes. Adhesion molecules are classified as either *selectins* or *integrins*. Apart from their role in leukocyte homing, integrins also play a role in maintaining the proper structure of the periodontal ligament (22). Interestingly, the epithelial integrin αvβ6 participates in homeostasis of the lungs by activating the immunosuppressive cytokine TGF-beta, and thus restraining the activation of alveolar macrophages (23). A similar effect of integrins can be expected in the periodontal environment, where a hyper-responsive inflammatory response is a key mechanism for the tissue destruction occurring in periodontitis. Selectins can be divided into three family members: *P-, E-*, and *L-selectin*, based on the cell type on which they were identified: platelet, endothelium, and leukocyte, respectively. They mediate leukocyte rolling in response to specific activation signals from C5a, interleukin-1β, or TNF-α. The integrins bind to endothelial intercellular adhesion molecules ICAM-1 and ICAM-2, favoring the transendothelial migration of leukocytes (24). The β_2_-integrins (CD18) play a role specifically for PMNs: their extravasation, and during phagocytosis and the respiratory burst (24).

Adhesion molecule deficiencies can lead to severe infection, leukocytosis, and rapidly progressive periodontal disease in humans (25). In a study by Baker and coworkers (11), the role of adhesion molecules in the onset of alveolar bone loss was analyzed in adhesion molecule deficient mice. They used three strains of mice lacking or with severe reduction of β_2_-integrin *Cd18, Icam-1*, and *P-selectin*. Despite the absence of an exogenous infection (e.g., *via P. gingivalis)*, both the *Icam-1* and *P-selectin*-deficient mice were more susceptible to alveolar bone loss than WT mice. A recent study using *Lfa-1/Cd18* knockout mice demonstrated that increased degradation of alveolar bone was associated with increased local production of IL-17 (26). Blocking of IL-17 or its associated IL-23 decreased periodontitis progression in *Cd18* knockout mice and caused lower levels of pro-osteoclastogenesis cytokines IL-1β and RANKL. This inhibition also dramatically influenced the composition of the inflammatory infiltrate of the periodontium: lower numbers of CD3- (general T-cell marker), CD4- (specific subset of T-cells), and CD138- (plasma cells) positive cells were observed. This study showed that the inability of PMNs to migrate into the inflamed periodontium causes an influx of other immune cells and causes Th17 reactions that lead to periodontal destruction, resembling leukocyte adhesion deficiency-I type periodontitis seen in humans (26). Interestingly, blocking of IL-17 caused a reduction in the total bacterial burden, suggesting that the IL-17-driven inflammation contributed to the microbial dysbiosis, which in turn caused more periodontal destruction. A different mouse model, a knockout of the β6 integrin, resulted in a higher presence of bacteria in the periodontium, and consequently in periodontitis upon infection with periopathogens (27).

Similarly, Niederman et al. (28), who used a bacterial induction of periodontitis, showed that *P-* and *E-selectin*^−/−^ mice experienced significantly more alveolar bone loss than the WT counterpart. Bone loss occurred at an earlier age and was also accompanied by a 50-fold increase in the total gingival bacterial load in the knockout mice. Moreover, a highly significant correlation between the extent of bone loss and the total bacterial burden in the *P/E*^−/−^ group was observed. These knockout mice presented with a leukocytosis that resulted from the inability of the PMNs to transmigrate from the vasculature into the tissues.

**Figure 2:**
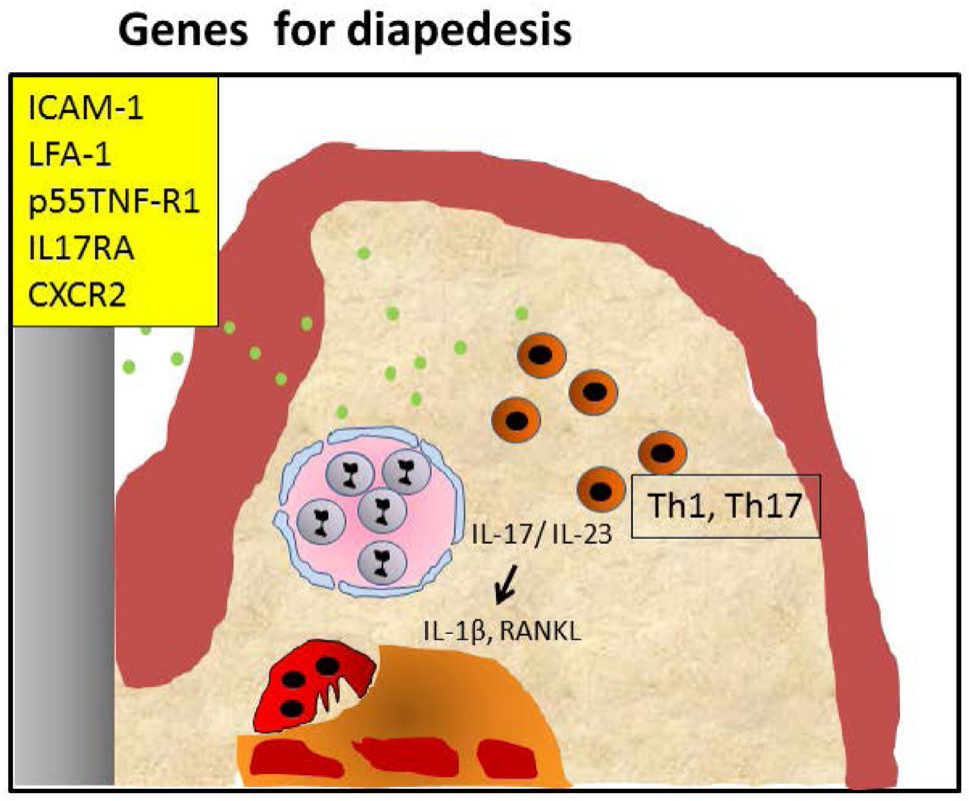
Periodontitis caused by malfunctioning diapedesis. Knockout of leukocyte adhesion molecules such as ICAM-1, LFA-1, and P-selectin and mice with defective TNF-α receptor p55 tumor necrosis factor-α receptor (p55TNF-R1), IL-17RA, or CXCR2 mediated attraction of polymorphonuclear neutrophils (PMNs) causes a diminished penetration of PMNs in the infected periodontium, resulting in hampered clearance of periopathogens (green dots). This may lead to an accumulation of Th1/Th17 cells, both in humans and mice. IL-17 and IL-23 activate alveolar bone loss by increasing pro-osteoclastogenesis cytokines IL-1β and RANKL. This sequence of events can be blocked by anti-IL-17 (26).

These data together show that the susceptibility of mice carrying adhesion molecule deficiency in different degrees is primarily related to the inability of the PMNs to exert their role in the initial phase of the inflammatory process, and to a secondary Th17-driven dysbiosis with exacerbated osteoclastic activity. We have summarized these events in Figure 2.

### Recognition and Killing of Bacteria: Toll-Like Receptors (TLR2 and TLR4), LAMP-2, and Lactoferrin

Toll-like receptors (TLRs) are pattern recognition receptors that recognize bacterial and viral compounds which stimulate innate immune responses (29). They are expressed by various cell types (epithelial cells, monocytes/macrophages, fibroblasts, and PMNs). When the TLRs interact with microbial components (e.g., LPS, fimbriae), they are activated and trigger the nuclear translocation of nuclear factor-κB (NF-κB) factor and induction of inflammation-related genes (30). In particular, this pathway leads to the production of immune mediators that initiate the inflammatory response against the microbial challenge. The TLR superfamily includes different classes of molecules and some of them have been investigated in relation to experimental periodontitis.

Burns et al. (31) analyzed the response of *Tlr2* deficient mice to a challenge with live *P. gingivalis*. The infected WT mice had more alveolar bone loss than the uninfected WT mice. Unexpectedly, the *Tlr2*^−/−^ mice were protected from *P. gingivalis*-induced bone loss. Using confocal microscopy and fluorescence-activated cell sorting, they showed that clearance by PMN-mediated phagocytosis of *P. gingivalis* in the absence of TLR2 was *more* efficient compared to WT mice. Moreover, wild-type mice showed a higher inflammatory cytokine (IL-1β and TNF-α) production than the *Tlr2* knockout mice. This suggests that in the presence of TLR2, the emerging cytokine milieu is sustaining a pro-inflammatory state, resulting in a favorable ecosystem for *P. gingivalis* survival, maintaining dysbiosis thereby worsening of the periodontal condition.

The involvement of different TLR types in periodontal bone loss varies across mice strains, depending on their genetic make-up. Costalonga et al. (32), using C57BL/10J, BALB/cJ and C57BL/6J mice, showed various effects of *Tlr2* or *Tlr4* deficiency. The *Tlr4*-deficiency worsened the periodontitis in the C57BL/10J mouse, but not in the BALB/cJ mouse. In the same study, the authors showed that the C57BL/6J TLR2 knockout mice had comparable alveolar bone levels when they were either sham- or *P. gingivalis*-infected. Thus, when interpreting the studies (25, 26), one must keep in mind the variability in the cytokine response elicited by a microbial challenge in the mice studied. The BALB/c mice tend to respond with Th2 cytokines (e.g., IL-4, IL-10, IL-13) and develop more bone loss, whereas the C57BL/6J mice produce predominantly Th1 cytokines (e.g., interferon-γ, IL-2) and are protected from periodontal bone loss. Therefore, ablating TLR2 or TLR4 signaling, modifies differentially the susceptibility to develop periodontitis and dependent on mouse strain. In contrast to the findings with *P. gingivalis*, TLR2^−/−^ mice that were infected with *A. actinomycetemcomitans* developed periodontitis (33). This suggests that the requirement for TLR2 to combat periodontopathognic bacterial products depends on the specific bacterial species.

LAMP-1 and LAMP-2 are two major lysosomal membrane proteins crucial for the protection of the lysosomal membrane from the host intra-lysosomal environment. LAMP proteins, especially LAMP-2, are important regulators in successful maturation of both autophagosomes and phagosomes (34). LAMP-2 is essential for the process of fusion between phagosome and lysosome that leads to the creation of a phago-lysosome in the PMNs. The phago-lysosome formation is a prerequisite for the successful degradation of internalized pathogens (35). Beertsen and coworkers (6) used a knockout mouse model of *Lamp-2* to investigate the role of this membrane-associated protein in phagosomal maturation. *Lamp-2* knockout mice experienced more bone loss already at 7 weeks after birth than the wild-type group without any exogenous bacterial infection. The bone loss was associated with a massive plaque accumulation on the tooth surface and large infiltrated epithelial areas in *Lamp-2* deficient mice. Interestingly, inflammation completely disappeared after applying antibiotics. Electron microscopic analyses of PMN revealed that these phagocytes isolated from the *Lamp-2*^−/−^ mice contained an accumulation of autophagic vacuoles due to the impossibility for the phagosomes to fuse with the lysosomes. This study underlines the importance of the PMN in functioning as the first line of defense; the PMN have the capability for oxygen-independent killing of bacteria to prevent the onset of periodontal disease and to protect against bacterial invasion and thus to avoid the generation of a pro-inflammatory state which is favorable for development of a dysbiosis.

Lactoferrin is an antimicrobial protein that has the capacity to reduce viability and pathogenicity of invading microorganisms using its properties to scavenge free iron from fluids and tissues. PMNs are the main source of lactoferrin delivery at the front of the bacterial biofilm facing periodontal tissues (36). *In vitro* studies show that lactoferrin has also the property to inhibit osteoclast differentiation (37). *Lactoferrin* knockout mice have been used to investigate the occurrence of alveolar bone loss in case of *A. actinomycetemcomitans*-induced periodontal disease (38). *Lactoferrin*-deficient mice experienced significantly more alveolar bone loss than wild-type littermates. The increased susceptibility of the *Lactoferrin*^−/−^ mice was also associated with an increased tissue level of *A. actinomycetemcomitans* and increased levels of IL-1β, IL-6, TNF-α, INF-γ, and IL-12 and chemotactic cytokines like CXCL10, involved in leukocyte migration. Thus, the *Lactoferrin* knockout mouse model clearly shows that lactoferrin is important in the prevention of alveolar bone loss induced by one of the major periodontitis-associated bacteria.

**Figure 3:**
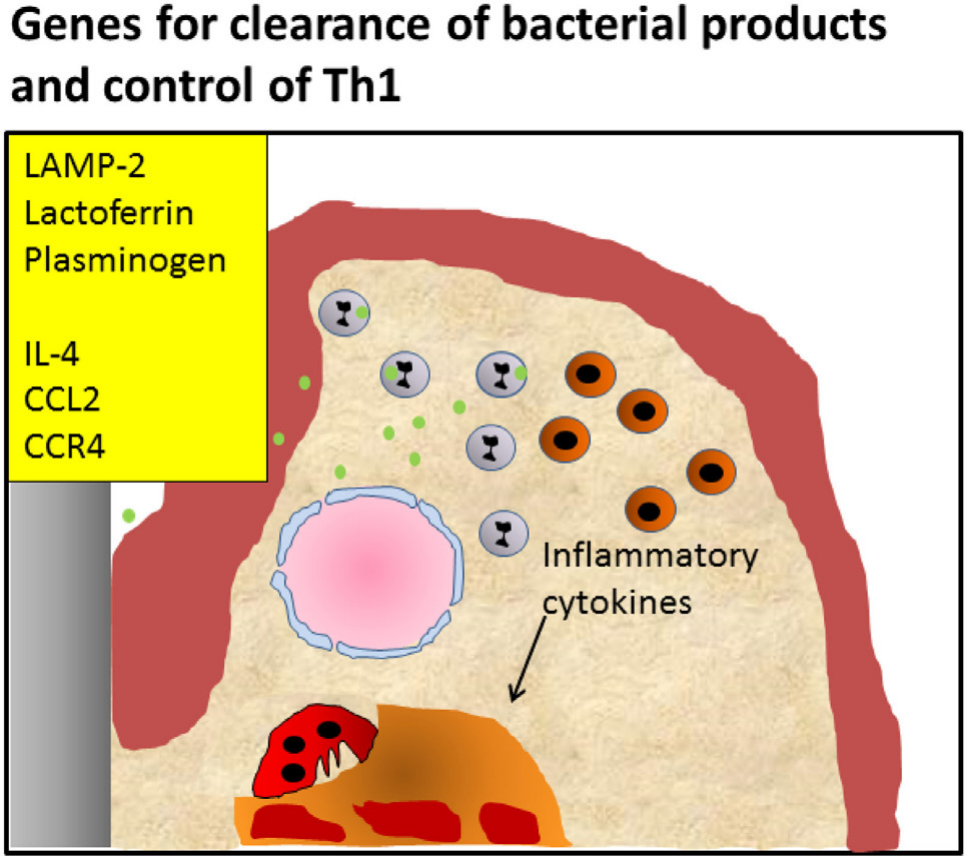
Periodontitis caused by defective killing of bacteria. Knockout of pattern recognition receptors such as Toll-like receptors, knockout of lysosomal membrane protein LAMP-2, antimicrobial protein lactoferrin, or plasminogen causes defective clearance of bacteria, leading to activation of the Th1 signaling pathway, and initiating chronic inflammation Alternatively, knockout of genes important for the induction of the Th2 reaction and influx of T regulatory cells such as IL-4, CCL2, and CCR4 that modulate the infection, also lead to enhanced inflammation and an endured Th1 presence, such as shown by Araujo-Pires et al. (8). It can be envisaged that bacterial products accumulate due to ineffective clearance.

Taken together, the deficiencies in *Tlr, Lamp-2*, and *Lactoferrin* illustrate the emerging inflammatory reactions when the innate immune mechanisms fail to effectively deal with microbial challenges. In fact, in the case of LAMP-2, the break of tolerance to naturally occurring dental biofilms is apparent. Thereby the Th1 inflammatory reaction is sustained, opening the way to chronic periodontal inflammation, a Th2 response and ultimately to bone degradation (Figure 3).

### Immune Modulation: Chemokines, Cytokines, Growth Factors, and Transcription Factors

The host response in the periodontal tissues to the bacterial biofilm on the teeth involves the release of chemokines, cytokines, and growth factors that chemo-attract, activate, and/or inhibit the local cells and the immune cells. In the normal (i.e., tolerant) situation, this plethora of modulators results in a stable balance without obvious inflammation. When for some reason the host response converts to an aberrant or hyperactive state, the combined action of all modulators initiates first of all innate immune response. If this initial response is not resolved, a switch to a more acquired immune response, where a tailor made specificity by B- and T-lymphocytes is achieved. What happens in terms of periodontitis progression when one or more of these immune modulating molecules is missing, is listed below.

#### Suppressor of Cytokine Signaling-3

Mice with a null-mutation of *Socs-3*, the “Suppressor of Cytokine Signaling” (SOCS)-3 proteins, develop periodontitis when they are infected with *P. gingivalis*. In this specific experimental periodontitis mouse model, the investigators have noted increased expression of RANKL and an increase in osteoclast activity (39); moreover, in line with the function of *Socs-3*, they showed an increased expression of pro-inflammatory cytokines such as IL-1β and IL-6.

#### SMAD2 Overexpression

SMAD2 is a transcription factor of the TGF-β signaling pathway. Using a mouse model with overexpression of SMAD2 in epithelial, but not connective tissues, Alotaibi et al. (40) showed that transgenic mice were highly susceptible to alveolar bone loss, compared to their wild-type controls. The mechanisms behind this phenomena is that SMAD2 induces an increased expression of TGF-β in the gingival junctional epithelial cells, resulting in higher TNF-α and RANKL levels, and more osteoclasts in the periodontium. However, the authors note that *Smad2* transgenic mice also showed reduced proliferation of the junctional epithelium in conjunction with increased apoptotic rates, resulting in a reduced surface area of the junctional epithelium. This suggests that the observed periodontal bone destruction in the *Smad2* transgenic mice could be the result of a reduced epithelial barrier function combined with a heightened activity of the TNF-α-RANKL-osteoclast axis.

#### CCL3, CCR1, and CCR5

The chemokine CCL3 (also known as macrophage inflammatory protein-1α, MIP-1α) binds to the chemokine receptors CCR1 and CCR5 primarily expressed on macrophages, dendritic cells, osteoclast precursors, and Th1 lymphocytes. These interactions result in their chemoattraction, activation, and their production of cytokines. As both macrophages and Th1 cells can stimulate the formation and activity of bone resorbing osteoclasts through TNF-α and INF-γ production, the axis CCL3/CCR1/CCR5 might be relevant for periodontitis pathogenesis. Repeke and coworkers employed mouse models with target deletion of *Ccl3, Ccr1*, or *Ccr5* (41). When challenged by *A. actinomycetemcomitans*, the *Ccl3*^−/−^ mice showed comparable periodontal destruction to the WT. The authors explained these findings by the presence of redundancy in the chemokine system, since the CCL4 and CCL5 can compensate for the lost functions of CCL3. Ablation of *Ccr1* or *Ccr5* resulted in reduced leukocyte infiltration, and this protected the mice from bone loss. Importantly, the protective effect was stronger when both receptors were knocked out simultaneously, suggesting a cooperative role for these chemokine receptors. These results suggest that an exaggerated inflammatory response is the main modulator of periodontal bone resorption.

#### IL-17RA, CXCR2, IL-4, and CCL22/CCR4

IL-17 is secreted by a subset of T-helper cells called Th17 cells. Its release is stimulated by TGFβ, IL-6, and IL-23 (42). IL-17 is involved in PMN recruitment and both in bone turnover, being modulatory in both bone formation and bone degradation (43), the latter especially in inflammatory diseases such as rheumatoid arthritis (44). IL-17RA is a main receptor of IL-17 and its activation by IL-17 generally results in the activation *via* the activation of NFk-B pathway to produce other pro-inflammatory cytokines (45). Yu et al. (10) investigated the role of IL-17RA in alveolar bone loss after an infection with *P. gingivalis*. With the knockout of *IL-17ra* the activity of IL-17 activity is disrupted. A morphometric analysis showed that *IL-17ra*^−/−^ mice challenged with *P. gingivalis* had significant more bone loss at multiple molar sites (from 29 to 57%) in comparison with infected wild-type mice. Thus, the absence of the signal-modulating process mediated by IL-17RA results in bone loss after infection with *P. gingivalis*. Notably, alveolar bone loss was explained by an impairment in the PMN’s migration toward the gingiva in *P. gingivalis*-infected *IL-17ra*^−/−^ mice: the PMN response at the site of gingival infection was reduced, as revealed by PMN counts in tissue sections. The failure in PMN migration is a consequence of inadequate levels of recruitment-related chemokines CXCL5 and growth related protein-α (Groα). In the same study and under the same experimental circumstances (10), *Cxcr2*^−/−^ mice showed significantly more alveolar bone destruction than WT mice with an even more severe phenotype than *IL-17ra*^−/−^ mice in terms of bone loss. CXCR2 is a receptor expressed on the surface of PMNs, it binds IL-17-induced chemokines like CXCL5, Groα, and macrophage inflammatory protein 2. These molecules are CXC chemokines and they play a deterministic role in the recruitment of sufficient PMN and therefore in alveolar bone conservation in the wild-type mice. We interpret the findings that most likely the chemokines regulate a normal and tolerant immune response.

Mouse models are particularly useful to shed light on the successive invasion of immune cells during periodontitis progression and the consequences for alveolar bone levels when certain immune cells cannot migrate into the periodontium. Araujo-Pires and coworkers (8) showed that when alveolar bone loss progresses rapidly after infection with a periodontal pathogen, it is accompanied by an initial influx of Th1 and Th17 cells, which leave the periodontium when the disease becomes chronic. Notably at these stages, Th2 (IL-4+) and Tregs migrate into the periodontium and slow down disease progression (Figure 1A). The Th2 and Treg cells express CCR4 and mice that lack this chemokine exhibit impaired influx of Tregs, accelerated bone loss accompanied with increased expression of pro-osteoclastogenic cytokines RANKL, IL-6, IL-17, and TNF-α and decreased expression of anti-osteoclastogenic cytokines osteoprotegerin (OPG), IL-10, and TGF-β. Interestingly, the rapidly progressive bone loss as well as the altered expression of pro- and anti-inflammatory cytokines could be reverted when the mice were injected with Tregs. In the same study, CCL22 could be identified as the chemokine that is important in the attraction of Th2 and Tregs. Mice treated with CCL22 neutralizing antibodies exhibited less Tregs concomitant with more alveolar bone loss. The investigators further show that CCL22 expression was severely limited in *IL-4* knockout mice. These mice were a phenocopy of the *Ccr4* null mutants: more bone loss, concomitant with more pro-osteoclastogenic cytokines. The phenotype could again be rescued by injecting Tregs (8).

The crucial experiments outlined above, showed that IL-17RA, CXCR2, IL-4, and CCL22/CCR4 are required for the natural sequences of influxes Th1, Th2, and Tregs in the periodontium. Loss of these moieties causes an enduring Th1 response by preventing Tregs to migrate into the periodontium.

#### p55 Tumor Necrosis Factor-α Receptor (p55TNF-R1)

TNF-α promotes recruitment of leukocytes *via* chemokine upregulation and production of matrix metalloproteinases (MMPs), needed for migration into the tissues, and mediates a wide range of inflammatory and antimicrobial effects through the TNF-α receptor p55 abbreviated as (p55TNF-R1) (46). TNF-α is recognized as an important mediator in periodontitis and its levels are increased in gingival crevicular fluid (GCF) of patients (47). The *p55Tnf-r1* knockout mice showed significantly less severe bone resorption in comparison with wild-type mice after an oral infection with *A. actinomycetemcomitans* (48). This was also accompanied by a mild inflammatory reaction given the significantly reduced number of leukocytes in the knockout group. The compromised PMN migration is dictated by the lower expression of PMNs chemoattractants (CXCL3, CXCL1, and their receptor CXCR2) in the *p55Tnf-r1* knockout mice. These chemokines are analogs of human IL-8, involved in PMN chemoattraction. Moreover, increased levels of IL-10, OPG, MMPs, and RANKL mRNA expression were seen in the *p55Tnf-r1* deficient mice in comparison to the wild-type group. The bacterial load of *A. actinomycetemcomitans* was increased in the *p55-Tnf-r1* knockout mice, indicating that this TNF-α receptor is important for proper clearance of bacteria. It is concluded by the authors that impaired TNF-α–p55TNF-R1 signaling causes protection against periodontitis through dampening of PMN invasion, hereby likely attenuating the osteoclastogenic response, despite a higher bacterial pressure.

#### IL-1RA

Like TNF-α, IL-1 is also a key cytokine in men and mice, and produced at any inflammatory process. It was first discovered as a bone resorbing cytokine and is known to activate osteoclasts (49). IL-1 receptor antagonist (IL-1RA) binds to the IL-1 receptor and prevents IL-1 signaling. Thus, mice lacking this regulatory protein may have a sustained activity of IL-1. Izawa et al. (50) compared periodontitis susceptibility after a challenge with *A. actinomycetemcomitans* between controls and *IL-1ra* deficient mice. Periodontitis was only established in the infected IL-1RA deficient mice, concomitant with increased formation of osteoclasts. Strikingly, no signs of periodontitis were observed in the control mice after infection, nor in the *IL-1ra* knockout mouse without bacterial infection. This indicates that a bacterial stimulus together with a sustained IL-1 signaling is needed for periodontitis progression. Expression of IL-1RA increased in *A. actinomycetemcomitans* infected WT mice increased numbers of osteoblasts, indicating that the organism activates its own negative feed-back loop after bacterial challenge.

In a different, transgenic mouse model with upregulated IL-1 signaling, the effects of IL-1α overexpression in oral epithelial cells was studied without an added bacterial challenge, just with the resident microbiome of the mice under study (51). These mice developed severe periodontitis that had all the characteristics of human periodontitis (loss of epithelial attachment, periodontal pocketing, and destruction of alveolar bone). Importantly, the total bacterial burden did not differ between the transgenic mice and their wild-type littermates. Taken together, these findings support the notion that IL-1 is a key mediator in periodontitis pathogenesis and suggest that IL-1 is certainly an important therapeutic target in human periodontitis.

#### IL-10, IL-12p40, and Stat3

IL-10 is one of the most important cytokines with anti-inflammatory properties (52). It is produced by activated immune cells, especially monocytes/macrophages and T cell subsets (e.g., Th1 cells). In an autocrine fashion in monocytes/macrophages, IL-10 diminishes the production of inflammatory mediators and inhibits antigen presentation, though it enhances the uptake of antigens (53). IL-10 plays a role in the immunopathogenesis of chronic inflammatory diseases including periodontal disease (54).

When *IL-10* knockout mice were infected with *P. gingivalis via* oral lavage, *IL-10*^−/−^ mice exhibited three fold more bone loss in comparison with WT mice after infection with *P. gingivalis*. This effect did not appear to be mediated *via* IL-1 since a neutralization of IL-1α, IL-1β, and IL-1RI with antibodies directed against these cytokines and receptor did not temper bone loss. This increased alveolar bone loss was not associated with an increase in the bacterial load in terms of CFUs that were grown after harvesting from the gingival crevice, and was comparable with the wild-type group. Thus, IL-10 seems to play a protective role. This is in agreement with Al-Rasheed et al. (55) who showed that a higher level of alveolar bone loss was evident in *IL-10*^−/−^ mice compared with *IL-10*^+/+^ mice, albeit that here, no bacterial infection had been introduced. A possible explanation of the *P. gingivalis*-induced bone loss in *IL-10*^−/−^ mice can be found in the paper of Sasaki and collaborators (56), who looked at the IL-10 downstream signaling molecule Stat3. By making use of different knockout mouse models, they found that macrophages/PMN-specific *Stat3*-deficient mice exhibited more alveolar bone loss than T cell- and B cell-specific *Stat3* mice, which were resistant to alveolar bone loss. This study indicated that both the monocyte/macrophage and the granulocytic (especially the PMN) lineages are targets for the immunosuppression by IL-10. Also the *IL-12p40/IL-10* and T cell/*IL-10* double deficient mice showed resistance to alveolar bone loss in comparison to *IL-10* single knockout mice. These data strongly suggest that the T cell responses mediated *via IL-12p40* stimulate alveolar bone destruction in an *IL-10* deficient state. Interestingly, Sasaki et al. (56) showed that the prophylactic or therapeutic treatment of *IL-10*^−/−^ mice with anti-inflammatory 18β-glycyrrhetinic acid (GA) can completely inhibit *P. gingivalis*-induced alveolar bone loss in mice, indicating that the anti-inflammatory mode of action of IL-10 is needed to prevent periodontitis. The *in vitro* analysis of resident peritoneal macrophages isolated from *IL-10*^−/−^ mice after *E. coli* LPS challenge revealed that GA suppressed the production of IL-1β and IL-12p70 in a dose-dependent manner and also the RANKL-stimulated osteoclastogenesis was dramatically reduced by GA. The mechanism by which GA can inhibit alveolar bone loss seems to be related to its capacity to inactivate the phosphorylation of NF-κB *in vitro*.

#### IL-18 Overexpression

IL-18 is a member of the IL-1 family and can induce production of both Th1 and Th2 cytokines. Mice overexpressing IL-18 in the gingival tissues, develop periodontal destruction after being infected with *P. gingivalis* (57). The mechanisms of action of excess IL-18 in the gingiva appear to be T-cell mediated, as the NF-κB and RANKL levels were increased in the transgenic mice after *P. gingivalis* infection, whereas the interferon-γ was decreased.

#### OPG Knockout, RANKL Overexpression, and RANK Overexpression

Osteoprotegerin is the molecule expressed by osteoblast lineage cells, which inhibits osteoclast differentiation. OPG binds to RANKL complex and thus prevents the RANKL-RANK signaling to osteoclast precursor cells necessary for proper osteoclast differentiation (58). The RANKL to OPG ratio in periodontal tissue of periodontitis patients can be an indicator of alveolar bone loss (59). Koide and coworkers (60) investigated the effect on alveolar bone loss in *Opg* knockout mice. Knockout mice present with significantly more alveolar bone loss (twofold more) than the WT counterpart, occurring without any experimental bacterial application. An increased number of osteoclasts was observed in the alveolar bone compartment. In the same study, also the effect of RANKL overexpression on periodontitis development was assessed using a *Rankl* transgenic mouse. Remarkably, these mice did not develop periodontitis, but a lower bone density of alveolar bone was apparent (60). These observations indicate that disturbance of the naturally high levels of OPG relative to RANKL that normally prevail in the periodontium (59) results in destruction of alveolar bone.

Apart from knocking-out *Opg*, a similar interference with the RANKL-RANK-OPG balance can be achieved by overexpressing RANK. Mice that lack *Rank* are osteopetrotic with an overall lack of osteoclasts (61). Recently, it was shown that *Rank* transgenic mice develop periodontitis in the absence of external bacteriological pressure, likely due to an exuberant RANK-RANKL signaling (62). Apart from the apparent alveolar bone loss, these mice also display root resorption, thickening of the junctional epithelium and significantly more rests of Malassez, epithelial groups of cells within the periodontal ligament (62).

#### NF-κB Inhibition in Osteoblasts

Inflammatory cytokines and TLR signaling activate NF-κB, which in turn affects the function of osteoblasts and osteoclasts. Pacios et al. tested the NF-κB inhibition and bacteria-induced periodontitis in inhibitor of Kappa B kinase (*Ikk*) transgenic mice (63). Transgenic mice that express a dominant negative mutant of *Ikk*, which inhibits NF- κB in osteoblast lineage cells, are protected from alveolar bone loss in response to oral inoculation with *P. gingivalis* and *F. nucleatum*, in contrast to their wild-type counterparts. This effect was mainly due to enhanced bone formation by osteoblasts and reduced osteoclast numbers and activation, as the development of an inflammatory infiltrate containing PMNs and monocytes with consequent loss of connective tissue attachment were unaffected by the genetic manipulation. This study demonstrates that during inflammation, in addition to lymphocytes and monocytes/macrophages, osteoblasts are also a relevant source of RANKL, and thus are important in alveolar bone resorption.

### Proteases

A variety of proteolytic enzymes are involved in many processes. Relevant here are their involvement in the normal homeostatic remodeling of the periodontal supportive tissues including normal turnover and pathological degradation of alveolar bone. Proteases are also found in the systems that degrade bacteria and their pathogenic components. In the case of periodontitis, periodontal ligament and alveolar bone degradation can be excessive and can cause progressive breakdown of periodontal supportive tissue. Below we summarize the observations in three different protease knockout mouse models, those with the following genes knocked out: *Mmp-8, Cathepsin K*, and *plasminogen* in periodontitis mouse models.

#### MMP-8

MMP-8 (collagenase 2) as a collagenolytic enzyme is responsible for the pathological degradation of type I collagen, which is the predominant collagen type in the periodontal structures. Levels of MMP-8 are elevated in gingival tissue, GCF and saliva in periodontitis patients (64). MMP-8, highly expressed in neutrophils, also possesses anti-inflammatory properties because it is able to cut and thus inactivate anti-inflammatory chemokines and cytokines (65).

In a study by Kuula and coworkers (66), the role of MMP-8 in periodontitis was investigated using an *Mmp-8* knockout mouse model infected with *P. gingivalis*. *Mmp-8*^−/−^ mice were infected with *P. gingivalis via* oral lavage to induce marginal periodontitis. A histological analysis showed that bone loss was significantly increased in the *P. gingivalis*-infected *Mmp-8*^−/−^ group compared to the *P. gingivalis*-infected WT group. The authors conclude that MMP-8 plays a protective role in alveolar bone loss during periodontal infection, possibly by inactivating pro-inflammatory cytokines. These findings are in agreement with research conducted by Hernández et al. (67) using the same *Mmp-8* knockout under *P. gingivalis* bacterial pressure. Furthermore, these latter authors showed that the expression in the gingival papilla of LPS-induced CXC chemokine LIX/CXCL5, a potent PMN chemoattractant, was significantly higher in the *P. gingivalis-*infected WT group compared with both infected and uninfected MMP-8 knockout groups. LIX/CXCL5 can regulate the PMN influx to periodontal tissues. In clinical dentistry, however, elevated salivary MMP-8 levels have been proposed to be diagnostic for periodontitis (68), but large-scale validation studies are needed. Moreover, these findings are opposed to the findings regarding the role of MMP8 in mouse periodontitis; it could be suggested that MMP8 facilitates the primary immune reaction by enabling the influx of the appropriate immune cells. When not present, it may lead to an enduring inflammatory response, resulting in the defective clearance of bacteriological products.

#### Cathepsin K

Similar to the above described *Mmp-8* knockout mice, mice that lack expression of the osteoclast-related protease cathepsin K (69) are protected from developing bacterium-induced periodontitis (70). Unexpectedly, *Cathepsin K* deficiency led to an absent TLR expression in the gingival epithelium, suggesting that Cathepsin K may somehow influence the expression of TLRs. *Cathepsin K* deficient mice were protected both for developing rheumatoid arthritis and periodontitis (70). This study further showed that both DCs and macrophages express Cathepsin K and that these cells are found at a much lower density in the periodontium of cathepsin K deficient mice that were infected with a cocktail of periodontopathogenic bacteria. Likewise, the number of T-cells did not increase in the periodontium after an infection. *In vitro* cultured dendritic cells from *Cathepsin K* deficient mice had a tempered reactivity when triggered with typical TLR triggers LPS and the nucleic acid sequence CpG. Thus, these studies propose a new role for cathepsin K, i.e., as a modulator of the immune response. In a pre-clinical study, the same group has exploited this model by treating wild-type infected mice with odonacatib, an inhibitor of cathepsin K. Thus, they could pharmacologically achieve inactivity of cathepsin K. It was shown that odonacatib—a Cathepin K inhibitor that was withdrawn from the market due to side effects—treated mice were also protected against periodontitis (71). From these studies, it can be deduced that cathepsin K plays both an immune modulatory role in dendritic cells and macrophages and a role in resorption in ostoclasts. Compounds that inhibit cathepsin K activity could be potential drugs to be further explored in the treatment or prevention of periodontitis.

#### Plasminogen

Plasminogen is an inactive proenzyme that is synthesized mainly in the liver (72). It is activated after cleavage into the serine protease plasmin. The activation can occur either *via* tissue-type plasminogen activator (tPA) or urokinase-type PA (uPA) (73). Plasmin also plays an important role in ECM remodeling because it degrades ECM components (e.g., laminin, fibronectin, proteoglycans) and activates MMPs (74). Plasmin may be important for host defense against infection (75). Indeed plasminogen deficiency has been associated to the onset of a destructive form of periodontal disease in humans named ligneous gingivitis/periodontitis (76). In a study by Sulniute and coworkers (77), the role of plasminogen in periodontitis was investigated using a *Plasminogen* knockout mouse model. Without additional bacterial infection, both the *tPa/uPa* double knockout—that cannot convert plasminogen into plasmin—and the plasminogen-deficient mutant mice, showed to develop periodontitis, as evidenced by alveolar bone loss: the plasminogen-deficient mice showed at any time point significantly more alveolar bone loss that increased with age up to 20 weeks compatible with a clinical picture of spontaneously developing periodontitis. At the histological level, this was associated with a massive PMN accumulation. Microbial analysis revealed a 100-fold increase in bacterial accumulation in plasminogen-deficient mice. One possible explanation is that phagocytic function of PMNs may be impaired in the absence of plasminogen (78). Interestingly, the systemic supplementation of human plasminogen in *Plasminogen*-deficient mice led to complete regeneration of soft periodontal tissues and significant regrowth of the alveolar bone. These results show that plasminogen is essential for a normal and tolerant host response in the periodontal tissues and prevents an aberrant, intolerant response to normal indigenous bacteria on teeth. Interestingly, genetic variants in the human plasminogen gene have not only been associated with atherosclerotic cardiovascular diseases, but were also associated with aggressive periodontitis in Northern European study populations (79).

### Mouse Periodontitis Models Involving Structural Alterations of the Periodontium

Alterations in formation and maturation of different compartments of the dental tissues have been linked to early-onset periodontitis in humans (80). This has been confirmed in several mouse models describing structural alterations in the periodontium and their associated periodontal destruction. Below, it is reiterated in all the mentioned studies that loss of integrity of the attachment of teeth to bone caused by loss of an important cementum or bone matrix protein, causes periodontitis.

#### Bone Sialoprotein (Bsp) Null Mice

Bone sialoprotein is an ECM protein present in bone, cellular, and acellular cementum (81). *Bsp*^−/−^ mice feature delayed bone and cementum growth and mineralization, but also progressive loss of periodontal tissues at later ages (82). The periodontal ligament in these mice loses its typical parallel and oblique fiber bundle orientation from root to alveolar bone and sparse periodontal ligament inserted in cementum. These results on the one hand reduced resistance to “pressure” from the epithelium allowing apical migration of the epithelium. Extensive root and alveolar bone resorption occurred in these mice, concomitant with increased RANKL expression. Likely, the disorganized fiber organization without tensile strength gives rise to RANKL expression and hence resorption. As reviewed by Sokos et al., the periodontal ligament usually protects against osteoclast formation by high OPG and low RANKL expression (59).

#### Dentin Matrix Protein 1 (DMP1) Null Mice

Dentin Matrix Protein 1 is another ECM protein, and is found in dentin, bone, cartilage, and cementum. *Dmp1*^−/−^ mice have, in addition to tooth abnormalities (enlarged pulp chambers, reduced dentin thickness) (83), also porous, hypomineralized alveolar bone and cementum, and a poorly organized PDL. As a result, *Dmp1*^−/−^ mice develop spontaneous early-onset periodontal breakdown, already when they are 3 months of age (84). Interestingly, the interdental bone shows mainly vertical defects, reminding of the localized early-onset (juvenile) periodontitis in humans. No attempt was made to control the outgrowth of microbiota in these mice, so a bacterial contribution to the observed periodontitis cannot be excluded, especially in older animals (up to 12 months). However, the authors noted that the vertical bone loss had occurred in the *Dmp1*^−/−^ mice as early as 3 months in the absence of overt signs of bacterial infection or inflammatory response.

#### Periostin

Periostin is a cell adhesion molecule and favors the cell–cell adhesion of pre-osteoblast attachment and spreading during bone formation (85). *Periostin*^−/−^ mice develop alterations of the PDL structure already at 4 weeks of age and later, at 3 months, they show radiographic signs of alveolar bone destruction coupled with a significant increase in osteoclast activity. The inflammatory response caused a replacement of periodontal ligament by granulation tissue as shown by the increased expression of collagen type III in the null mice (86). The apparent loss of cell–cell contact between periodontal ligament cells may alter the phenotype of these cells in the null mice into a more bone catabolic phenotype or compromise the barrier function of the periodontal ligament. The mice were maintained under specific pathogen-free conditions; however, they were not completely germ-free. The loss of the periodontal ligament barrier function might have created the conditions for a dysbiotic shift in the resident microflora of the *Periostin*^−/−^ mice, which might explain the massive PMN infiltrate in the affected periodontal tissues. Since Rios and coworkers (86) did not analyze the total bacterial burden in WT vs. null mice, a microbial pressure of resident species emerging in a dysbiotic state cannot be excluded as one of the contributing factors to the periodontitis that develops in the Periostin^−/−^ mice.

#### Dentin Sialophosphoprotein (DSPP)

Dentin sialophosphoprotein is expressed in dentin, bone, and cementum. DSPP mutations are associated with dentinogenesis imperfecta in humans. The corresponding murine model of dentinogenesis imperfecta is the *Dspp* knockout mouse. The *Dspp*^−/−^ mice have dental defects such as decreased cementum deposition (87). These mice show spontaneously alveolar bone loss as they age, comparable to periodontitis. Interestingly, mice that overexpress the NH_2_-terminal fragment of DSPP induces an even more severe periodontal phenotype in *Dspp*^−/−^ mice (88), indicating that this fragment has an inhibitory effect on the formation and mineralization of the hard tissues of the periodontium.

#### Ribosomal S6 Kinase

Coffin-Lowry is an X-linked genetic syndrome, characterized by mental and psychomotor retardation, skeletal and dental abnormalities. It is caused by mutations in the Ribosomal S6 kinase (RSK2), leading to complete inactivation of this enzyme. Dental abnormalities in humans include delayed eruption, hypodontia, and premature tooth loss. There is evidence from Rsk2-deficient mice, showing that the skeletal and dental defects are caused by impaired bone and cementum formation, respectively (89). At 4 months, the Rsk2-deficient mice showed hypoplastic and hypomineralized cementum, detachment of the PDL, apical migration of junctional and pocket epithelium concomitant with pocket formation and loss of alveolar bone. It was concluded that the premature tooth loss in Coffin-Lowry syndrome is most likely a consequence of defective cementum formation.

#### K14-VEGF Receptor 3-Ig (K14) Mice That Lack Lymphatic Vessels in Gingiva

In the gingiva, lymphatic vessels are normally found in the connective tissue layer below the oral and the junctional epithelium. These vessels widen during a bacterial challenge of the periodontium (90). The *K14-vegf receptor 3-Ig* transgenic mice lack overall lymphatics, including the gingiva. In response to oral inoculation with *P. gingivalis* develop alveolar bone loss than their WT littermates (91). The absence of lymphatics in the gingiva leads to a massive influx of macrophages around the alveolar bone, concomitant with an increased number of osteoclasts degrading the alveolar bone. A weaker activation of B cell-antibody production was also characteristic of this model. Levels of inflammatory cytokines were only increased in the infected *K14-vegf receptor 3-Ig* transgenic mice, suggesting that here periodontitis arose due to an enduring inflammatory response.

### Models on the Association between Periodontitis and Systemic Diseases

In humans, periodontitis often arises together with other inflammation-related diseases, so-called comorbidities. Development of genetic mouse variants that display both periodontitis and atherosclerosis or rheumatoid arthritis are useful in elucidating common denominators of these diseases. Here, we briefly review periodontitis/atherosclerosis and periodontitis/rheumatoid arthritis mouse.

#### Atherosclerosis

Periodontitis is linked to atherosclerotic cardiovascular diseases; in the last 20 years, hundreds of papers have emerged on this association (92) and plausible pathobiological mechanisms have been described (93). In addition to many epidemiological studies, also evidence on this association has been generated employing mouse models. The hyperlipidemic apolipoprotein (Apo) E-null mice have been used in a series of studies (27, 94, 95). By applying oral mono- or polymicrobial infections with *P. gingivalis, F. nucleatum, Treponema denticola*, and *Tannerella forsythia*, the authors showed that the ApoE^−/−^ mice develop not only destructive periodontitis, but also progressive atherosclerosis. The mechanisms involve systemic dissemination of periodontal bacteria, aortic bacterial colonization, skewed T cell polarization in the spleen, altered cytokine, and lipid profiles in mouse serum. Important to note, the emerging phenotype in polymicrobial infections was not the sum of responses to monoinfection with each microorganism, raising the issue of microbial synergism in periodontitis and pointing at microbe–microbe interactions as modifiers of microbe–host interactions.

The β6 integrin model knockout model is relevant to further strengthen the periodontitis–atherosclerosis relationship. Upon infection with periodontopathogens, these mice developed periodontitis simultaneously with atherosclerosis (27), as measured by lipid vesicle content of blood and of aortic wall. Several indicators were elevated, only in the infected knockout mice. This study shows that effects of periodontitis on the development of atherosclerosis have been neglected in nearly all periodontitis models described in this review.

#### Rheumatoid Arthritis

Hao et al. used a combined transgenic mouse model, the human transgenic *Tnf-*α- and *Cathepsin K*-deficient mice to study common pathogenic processes involved in rheumatoid arthritis and periodontitis (70). *Cathepsin K* deficiency was protective against both diseases, and the authors attribute that to the dampened inflammatory reactivity, with less TLR expression, less dendritic cells and less cytokines produced in the arthritis and periodontitis lesions. This hypothesis of the shared hyper-inflammatory phenotype in periodontitis and arthritis has been confirmed by Trombone and coworkers, in a model using the acute inflammatory reactivity maximum AIRmax and minimal AIRmin mice (96). The parallel induction of arthritis and experimental periodontitis with periopathogens (*A. actinomycetemcomitans* and *P. gingivalis)* in the inflammation-prone AIRmax mice resulted in a more severe phenotype: higher leukocyte infiltration, higher local levels of IL-1β, TNF-α, RANKL, IFN-γ, and IL-17, skewed T cell polarization toward Th1 and Th17, and more periodontal destruction. Interestingly, in this study, the presence of normal oral microbiota was essential for the induction of periodontitis. This finding identifies the exaggerated inflammatory phenotype as the enabler of the ecological shift from a commensal microbiota, which in standard homeostatic condition would not be harmful to the host, to a dysbiotic biofilm, incompatible with periodontal health, even in the absence of classic periodontopathogens.

## Concluding Remarks

### Mouse Models of Periodontitis—Mouse Models Human Periodontitis

When interpreting the diverse mouse models, it becomes apparent that single gene deletions can give rise to periodontitis. Some KO or transgenic mouse models show periodontitis developing with a normal resident oral microbiome, while most need an exogenous bacterial infection. These observations underscore the delicate balance of immune reactions that are needed in a sequential and efficient way to combat an infection. As indicated, one could classify mutations that give rise to periodontitis due to a malfunctioning infiltration or transmigration of immune cells such as PMNs into the periodontium challenged by bacteria in the sulcus (*LFA-1, ICAM-1, P-Selectin*). Likewise, infections can endure when Tregs are unable to migrate into the periodontium and modulate the infection due to critical modulators such as *IL-4, CCL2*, and *CCR4* (Figures 2 and 3). The second category is the defected clearance of bacteria or bacterial products of infiltrated but dysfunctional immune cells such as PMNs that are deficient in *LAMP-2, TLRs, lactoferrin*, or *plasminogen* (Figure 3).

### Periodontitis: A Second Hit Disease?

In many of the studies reviewed here, no periodontitis occurred in wild-type mice, not even after an infection with periodontopathogens. This was the case for 9 out of 12 studies where the fourfold comparison (wild-type; wild-type infected; knockout; knockout infected) was studied. Likewise, many of the knockout mice did not develop periodontitis in the absence of periodontopathogenic pressure (10 out of 11). Apparently, analogous to Bert Vogelstein’s famous second hit hypothesis for developing colorectal cancer (97), where two hits are required to develop disease (colorectal cancer), one could thus postulate that both an underlying genetic defect and a bacterial challenge are required for developing periodontitis. For seven genes: *IL17-ra* (10), *IL1-ra* (50), *Socs-3* (39), *IL-10* as well as its downstream modulator *Stat3* (55) and adhesion molecules *Icam-1* (11) and *Beta6 integrin* (27), only the combination of functional loss with bacterial pressure resulted in periodontitis.

Though this “second hit” hypothesis applies to the above models, where the combination of a genetic defect with periodontopathogenic pressure is required, it should be noticed that many models described here do not need this exogenous pressure to develop periodontitis. Examples are the *Lamp-2* (6), *Plasminogen* (77), *Opg* (60), *Rank* transgene (62). Peculiarly, it seems that this external pressure is not required in themutations that affect the structural integrity of the periodontium, such as *Bsp* (82), *Dmp1* (84), *Periostin* (86), and *Dspp* (87). Some of these mutations resemble genetic predisposition for developing periodontitis, such as seen in humans. We could thus make the distinction between genetic models that do require periodontopathogenic pressure and models where the mere genetic defect is enough to initiate periodontitis.

### Mouse Models Can Be Valuable for Developing Treatment Strategies

Genetic models for periodontitis may shed light on new treatment modalities. An intriguing example could be the lessons learned from the periodontitis resistant Cathepsin K knockout mouse. Apparently, interference with osteoclast function can prevent alveolar bone degradation. Besides that, the Cathepsin K knockout shed new light on the role of dendritic cells and subsequent immune cell influx, thereby modulation severity of the infection. Previously, it was shown that the periodontal status of rheumatoid arthritis patients receiving anti-TNF-α treatment (98) stabilized as a side-effect of treatment. A second example could be patients with defective CD11a/CD18 that are genetically prone to develop periodontitis. Moutsopoulos et al. have shown that mice and humans with this genetic defect have a highly enhanced presence and activity of Th17 cells in the periodontium. Mice with this defect develop periodontitis, which is blocked when treating these mice with anti- IL-17 antibodies (26). Treatment modalities interfering with IL-17 activity may thus be beneficial for the periodontal status of these patients.

## Author Note

Teun J. de Vries is a member of the Euroclast consortium (www.euroclast.eu), a Marie Curie Initial Training Network (ITN).

## Author Contributions

TV initiated the writing of this review. He wrote a large part of the manuscript and interpreted, summarized, and edited parts written by SA. TV made all the figures that interpret and summarize the literature. SA collected literature and did initial writing. BL contributed to two pre-final versions. He is the head of the department and a clinical periodontologist. His focus in this review was to further link mouse models and the clinic. EN has expertise in the field of granulocytes and wrote sections on DCs and other disease models that are comorbidities to periodontitis.

## Conflict of Interest Statement

The authors declare that the research was conducted in the absence of any commercial or financial relationships that could be construed as a potential conflict of interest.
